# Evaluation of WhatsApp as a Platform for Teledermatology in Botswana: Retrospective Review and Survey

**DOI:** 10.2196/35254

**Published:** 2022-07-27

**Authors:** Erika Koh, Abena Maranga, Tshepo Yane, Kagiso Ndlovu, Bwanali Jereni, Maitseo Kuno Nwako-Mohamadi, Carrie Kovarik, Amy Forrestel, Victoria L Williams

**Affiliations:** 1 Department of Dermatology Oregon Health & Science University Portland, OR United States; 2 Perelman School of Medicine University of Pennsylvania Philadelphia, PA United States; 3 School of Engineering and Applied Science University of Pennsylvania Philadelphia, PA United States; 4 Department of Computer Science University of Botswana Gaborone Botswana; 5 Department of Medicine University of Botswana Gaborone Botswana; 6 Princess Marina Hospital Gaborone Botswana; 7 Nyangabgwe Hospital Francistown Botswana; 8 Department of Dermatology University of Pennsylvania Philadelphia, PA United States

**Keywords:** dermatology, teledermatology, telehealth, eHealth, mHealth, WhatsApp, developing countries, Botswana, Africa, low income, retrospective review, instant messaging

## Abstract

**Background:**

In emerging market countries in sub-Saharan Africa, access to specialty services such as dermatology is limited. Teledermatology is an innovative solution to address this issue; however, many initiatives have been tried without sustained success. Recently, WhatsApp has been used as a store-and-forward telemedicine communication platform for consultation and education in Botswana.

**Objective:**

This study aims to describe the utilization of WhatsApp for teledermatology and the satisfaction levels of participating providers.

**Methods:**

A 2-part pilot study was conducted. First, a retrospective review was performed of WhatsApp communications received by participating dermatologists in Gaborone, Botswana, from January 2016 to December 2019. Sender information, patient demographics and history, response time, diagnoses made, and follow-up recommendations were collected. Second, a 12-question cross-sectional survey was distributed to health care providers who utilized WhatsApp for teledermatology during this period. Descriptive statistics were then performed.

**Results:**

There were 811 communication threads over the study period. The majority (503/811, 62%) of communications were consultations from providers inquiring about a specific patient, followed by multidisciplinary care coordination communications (90/811, 11%). Our in-depth analysis focused on the former. In 323 (64%) provider consultations, dermatologists responded within 1 hour. A diagnosis was made in 274 (55%) consultations. Dermatologists gave treatment recommendations remotely in 281 (56%) consultations and recommended an in-person dermatology visit in 163 (32%). Of the 150 health care providers surveyed, 23 (15%) responded. All respondents (100%) felt that there was a need for teledermatology and improved teledermatology education in Botswana. Moreover, 17 (74%) respondents strongly felt that the guidance received via WhatsApp was high quality, and 22 (96%) were satisfied with WhatsApp as a platform for teledermatology.

**Conclusions:**

This retrospective review and survey demonstrated that WhatsApp is a quick, well-received, and sustainable method of communication between dermatologists and providers across Botswana. The app may offer a solution to the challenges providers face in accessing specialty referral systems, point-of-care education, and medical decision-making support for complex dermatologic cases in Botswana. The information gained from this pilot study can serve as the basis for future telemedicine studies to improve the implementation of teledermatology in Botswana and other resource-limited countries.

## Introduction

Despite a high burden of dermatologic diseases, access to dermatologic specialty care is scarce in sub-Saharan Africa [1,2]. In Botswana, there are as few as 10 physicians per 100,000 people and even fewer dermatologists, all of whom are in large urban areas [3,4]. Currently, there are approximately 4 dermatologists practicing in the public health sector in Botswana to support a population of 2 million people. Primary care providers in Botswana have limited training in dermatology and face challenges in treating complex dermatologic conditions and successfully referring patients to specialists [5]. Additionally, coordinating care between specialties can be difficult in Botswana [6]. Thus, there is a significant need to improve the delivery of high-quality dermatologic care to remote settings by providing local health care workers with better access to dermatology expertise and education.

Teledermatology is a potential solution to address these challenges. Formal telemedicine platforms have been specifically designed to securely communicate predetermined sets of information between providers. Several have been developed and trialed in Botswana. In 2007, the Africa Teledermatology Project began providing health care providers in many sub-Saharan African countries, including Botswana, free access to a web-based platform for consultations, forum discussions, and educational materials [7]. In 2011, a partnership between the Ministry of Health of Botswana, the Botswana-UPenn Partnership (BUP), and the Orange Foundation of Botswana resulted in a multispecialty mobile telemedicine solution, including teledermatology, called “Kgonafalo” [8]. In 2015, through the Television White Space Project, several local and international partners collaborated to provide low-cost wireless broadband internet to improve telemedicine connectivity for remote clinics in Botswana [9]. However, sustained success has been difficult with these programs. The African Teledermatology Project, although still successfully running, operates primarily on a web-based platform, which can be difficult to access in remote locations. Technical challenges, such as limited desktop equipment, slow connectivity, and device malfunctions, are common. Kgonafalo utilized a specially developed mobile app, and the burden of training a constantly changing population of primary care providers was high. In addition, Kgonofalo used designated clinic mobile phones, which needed to be maintained and charged, and users needed to be comfortable using them. Most of all, these programs were difficult to implement due to loss of provider confidence and motivation to use formal telemedicine platforms in the face of multiple challenges [5,7,9]. 

Although formal teledermatology platforms can offer security and standardization, in low resource settings, the associated logistical and cost burdens frequently render them unfeasible or unsustainable, as previously seen in Botswana. Informal platforms are an alternative that allow the transmission of information via flexible, secure methods that can function on personal mobile phones and with lower bandwidth. Teledermatology through mobile health (mHealth) has demonstrated technical feasibility and reliability in providing care to underserved and remote populations around the world where smartphones are common, but the key to utilization is the ability to send consults within an app that is familiar to the user on their own mobile device [10-13]. mHealth was first introduced in 2009 in Botswana as a clinical education tool that was found to be effective and satisfactory among resident physicians [14]. In the past 10 years, studies have shown that mobile telemedicine systems are deemed acceptable by patients in Botswana [1] and have the potential to increase access to care across multiple specialties [5,8,15-18].

Mobile phone subscriptions have been increasing in resource-limited countries [19], and WhatsApp, a service with over 1 billion users worldwide, is the predominant form of electronic communication in Botswana [20]. In 2016, one of the authors (VW), who was working as a dermatology specialist in Botswana, noted the critical need for a sustainable method of teledermatology to connect providers across the country. In the absence of resources to develop and launch a new formal teledermatology program, she established a store-and-forward teledermatology consultation network using WhatsApp. Implementation was fast and easy because the application did not require dedicated training, specific equipment, or Wi-Fi connectivity, and most providers were already using WhatsApp for other types of communication [6].

Because WhatsApp is a relatively new platform for teledermatology, it is important to understand how physicians in Botswana are currently using it and gain user feedback to determine its feasibility, effectiveness, and potential to scale for use in other specialties. This pilot study aims to describe how the WhatsApp application is being utilized in Botswana to connect providers to dermatology expertise for patient care and education, as well as to elucidate current provider satisfaction with the platform.

## Methods

### Ethics Approval

This study was approved by the University of Botswana and the Botswana Health Research and Development Committee institutional review boards (HPDME 13/18/1) and was granted a review exemption by the University of Pennsylvania IRB (835003).

### Retrospective Review of WhatsApp Communications

#### Study Population, Setting, and Design

In the first part of the study, we conducted a retrospective review of teledermatology communications sent through WhatsApp in Botswana from January 2017 to December 2019. Messages from health care providers and patients seen within the public health care system of Botswana were sent to 2 dermatology consult mobile phones. Messages were reviewed by a full-time local dermatologist and rotating North American dermatology residents and faculty at Princess Marina Hospital (PMH) in Gaborone, Botswana. WhatsApp messages were downloaded from the mobile phones, and conversation threads found on both were identified as duplicates and removed. There was no specific record kept of which dermatologist reviewed and responded to individual messages.

#### Data Collection

An overview of the data extraction and categorization methods used to analyze conversation threads is summarized in Multimedia Appendix 1. Each conversation thread was broadly categorized by the purpose of the communication: consultation from a provider (nondermatologist to dermatologist), consultation from a patient (patient to dermatologist), remote patient management (inquiries regarding long-term management for known diagnosis), patient follow-up (provider message regarding patient already known to dermatologist), teletriage (requiring urgent dermatology appointment), multidisciplinary care coordination (organizing patient care activities between 2 or more providers), provider question (unrelated to a specific patient), and incomplete communication (lack of response from dermatologist, an abrupt stop in the conversation, or conversation continued outside of WhatsApp). Other purposes of communication were excluded. Sender information (phone number, profession, and location) was collected from all communications if available.

Further data collection was only performed on the consultations received from a provider. We extracted data into categories that were considered to be most important to patient care in Botswana, including patient demographics, history provided, photograph parameters, response time, diagnosis, and outcome of consultation. Patient age, sex, and HIV status were recorded if provided. The extent of a history of present illness (HPI) shared by the nondermatologist provider was based on a point system, with 1 point given for each of the following: description of the lesion, location on the body, symptoms reported, timing of onset, change in appearance over time, aggravating or alleviating factors, prior treatments performed, and pertinent lab or imaging results.

Photographs received with consultations were also reviewed. Two authors (TW and AF) with experience reviewing teledermatology consultation photos in Botswana developed a subjective grading system that rated photos as low, medium, or high quality based on criteria of image resolution, lighting, and content (whether photos captured relevant areas of the body). To standardize grading, photos were reviewed by 2 authors (VW and AF) until a consistent agreement on grading was achieved. After standardization was achieved, each photo was graded by 1 author. The file size (kB) of photographs was also recorded. Multimedia Appendix 1 provides more details of the process used to grade photographs.

Response times from dermatology consultants was stratified (0-60 minutes, 1-6 hours, 6-12 hours, 12-24 hours, 24-48 hours, and >48 hours) based on both the time from initial message sent to initial response and initial message sent to final diagnosis or recommendation. Dermatologists provided no diagnosis, a single diagnosis, multiple diagnoses, and/or differential diagnoses in response to consultations from providers. All diagnoses, including those that were differentials, were included in the overall analysis. Diagnoses were classified into the major categories of inflammatory disorders, infection, neoplasm, diseases of vasculature, and other diagnoses. Consultation outcomes were based on the dermatologist’s recommendation and divided into the following categories: advice for local management (when treatment recommendations were provided remotely), referral to see a dermatologist, referral to see a different specialist, or other recommendation. We recorded whether dermatologists provided education to providers (clinical information in addition to a diagnosis and treatment plan).

#### Statistical Analysis

Descriptive statistics were used to broadly categorize the conversation threads and demographic and clinical data provided in the consultations.

### Satisfaction Survey of Providers Using WhatsApp for Teledermatology

#### Study Population, Setting, and Design

In the second part of the study, we conducted a cross-sectional survey of health care providers in Botswana who used WhatsApp for teledermatology from January 2016 to December 2019.

#### Data Collection

A research electronic data capture (REDCap) survey was distributed via WhatsApp, and responses were kept anonymous. The target population was a convenience sample of providers that used the platform for consultations. Informed consent was obtained from all participants. This was a voluntary, open survey that consisted of 12 questions aimed at evaluating users' satisfaction and experience with the platform in terms of technical quality, perceived effectiveness and usefulness, privacy and security practices, and suggestions for improvements (Multimedia Appendix 2). We developed a novel survey that was not based on an existing validated survey instrument to evaluate for factors most pertinent within the local context. The first 6 questions used a Likert scale to evaluate the overall value of WhatsApp as a teledermatology tool. The subsequent 6 questions were multiple-choice questions regarding specific aspects of the platform as well as user practices. Survey questions were developed in REDCap by dermatology and informatics faculty at the University of Pennsylvania, Ministry of Health and Wellness of Botswana, and the University of Botswana, who had experience with the local health care system’s needs and limitations.

#### Statistical Analysis

The frequency of responses to survey questions were recorded and reported, and common themes in areas for improvement were identified.

## Results

### Retrospective Review of WhatsApp Communications

From January 2017 to December 2019, there were a total of 811 conversation threads, with 102 threads in 2017, 350 in 2018, and 324 in 2019. There were 35 threads with a missing date stamp that were also included in the analysis. Approximately 150 senders were identified based on unique phone numbers and names in the phone contact list. An exact number of senders could not be confirmed due to inconsistencies in the way contact information was saved in each mobile phone. 

**Figure 1:**
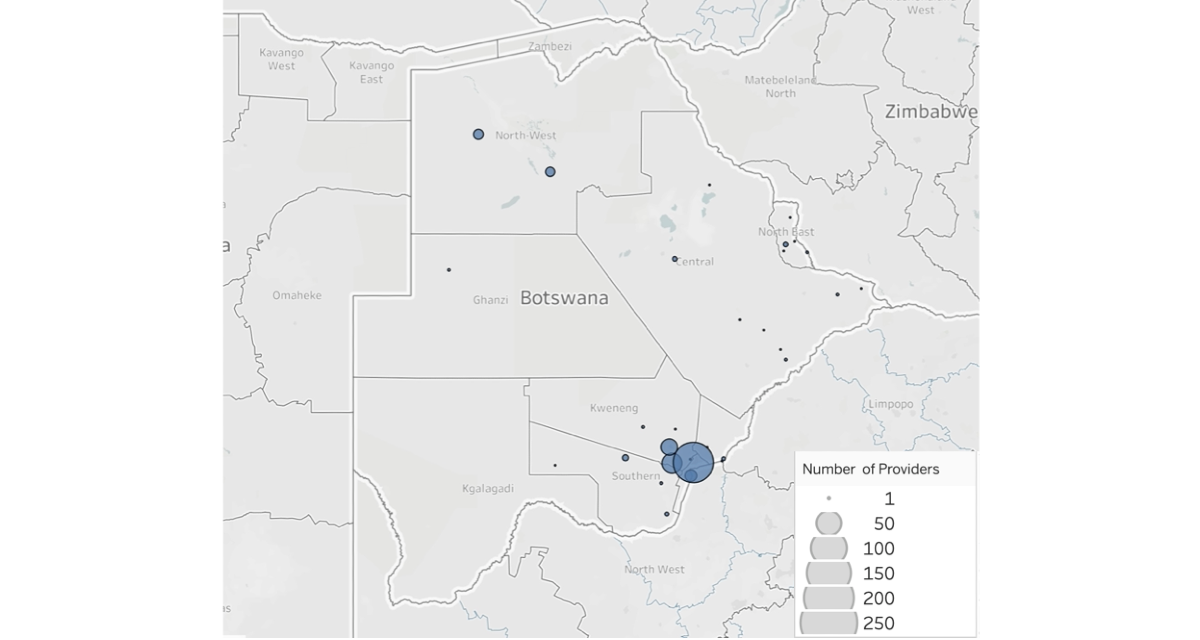
Locations of providers that utilized WhatsApp for teledermatology in Botswana, with number of providers in each location.

The most common (503/811, 62%) purpose of communication was a consultation from a provider, as seen in the conversation threads, followed by multidisciplinary care coordination (23/811, 11%) (Table 1). The profession of the provider was stated in 44% (355/811) of the conversation threads, and 90% (320/355) were physicians. The provider’s location was stated in 58% (473/811) of the threads. There was wide variation in locations across Botswana as well as other sub-Saharan African countries (Figure 1).

**Table 1:** Categories of communication between dermatologists and nondermatologist providers according to WhatsApp communication threads (N=811).

Purpose of communication	Communication threads, n (%)
Consult from provider	503 (62)
Consult from patient	23 (3)
Remote patient management	7 (1)
Patient follow-up	55 (7)
Teletriage	44 (5)
Multidisciplinary care coordination	90 (11)
Provider question	23 (3)
Incomplete consult	66 (8)

**Figure 2:**
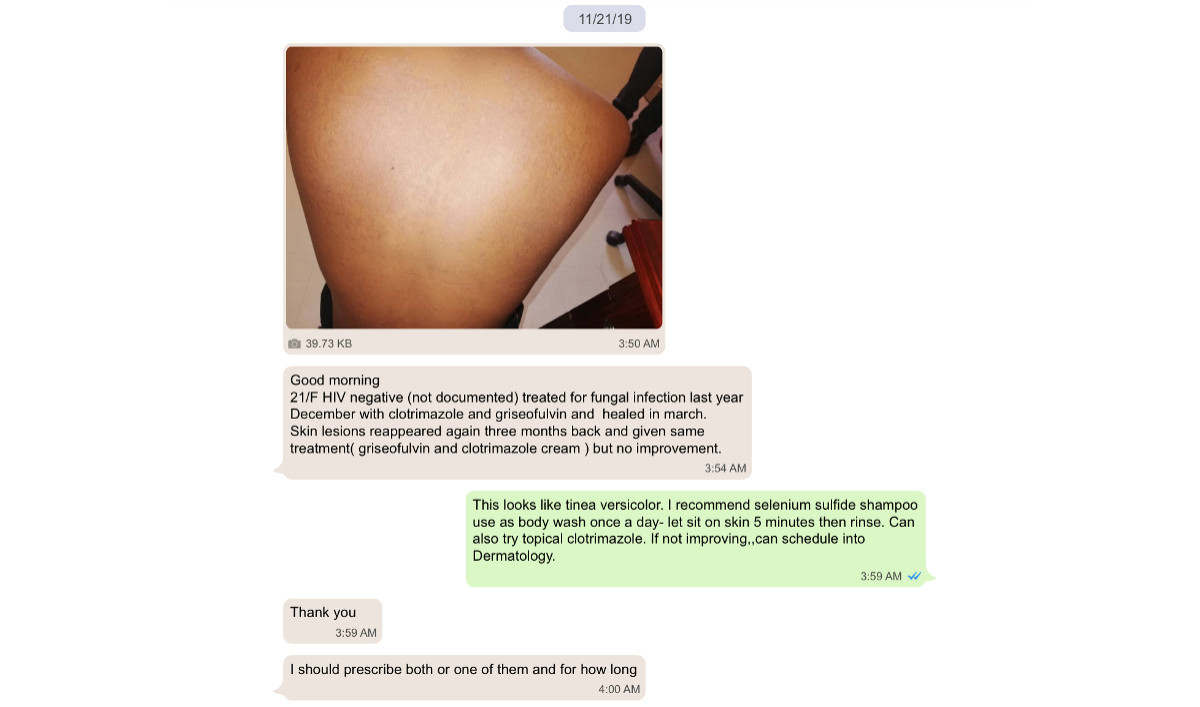
Example of a consultation sent from a nondermatologist provider to a dermatologist.

Our in-depth analysis focused on the 503 WhatsApp consultations from nondermatologist providers (Table 2). An example of a consultation is provided in Figure 2. Providers gave an average of 3.1 out of 8 possible points of HPI based on our point system. Patient age was provided in 76% (380/503) of the consults, sex in 76% (383/503), and HIV status in 47% (234/503). The majority (477/503, 95%) provided a photo. Responding dermatologists asked clarifying questions in 40% (200/503) of consults. The average patient consulted on was 30.5 years old (ranging 8 days to 84 years), in which 59% (226/503) were female, 41% (157/503) were male, and 38% (89/503) were HIV positive.

**Table 2:** Format of consultations sent by nondermatologist providers (N=503).

Measures			Consultations
Age provided, n (%)			380 (76)
Sex provided, n (%)			383 (76)
HPI^a^ provided, mean (SD)^b^			3.1 (1.6)
HIV status provided, n (%)			234 (47)
Photo provided, n (%)			477 (95)
**Photo subjective grade^c^, n (%)**			
	Low	101 (20)	
	Medium	198 (39)	
	High	178 (35)	
**Photo file size, n (%)**			
	<50 Kb	173 (34)	
	50-100 kB	214 (42)	
	100-150 kB	52 (10)	
	>150 kB	37 (7)	

^a^HPI: History of present illness.

^b^HPI provided in the consultation was graded on a point system, with 1 point given for each of the following: description of the lesion, location on the body, timing of onset, change in appearance over time, aggravating and alleviating factors, prior treatments performed, symptoms reported, and pertinent lab or imaging results.

^c^Subjective photograph quality was determined based on a grading system in which the criteria were image resolution, lighting, and whether relevant areas of the body were captured. Photos were graded on a scale of low, medium, or high quality based on the number of quality criteria met (Multimedia Appendix 1).

Dermatologists responded to the provider within 1 hour in 64% (323/503) of consultations and provided the final diagnosis or recommendation in 54% (272/503) (Table 3). In over half (274/503, 54%) of consultations, a single diagnosis or multiple diagnoses were made. A differential diagnosis was provided in 32% (159/503) of consultations. Dermatologists recommended management to be given by the local provider in 56% (281/503) of consultations, and in 32% (163/503), patients were recommended to schedule an in-person dermatology visit. Additional education was provided by dermatologists in 28% (140/503) of consultations.

Dermatologists provided 224 unique diagnoses out of a total of 704 diagnoses made. The most common were eczema, contact dermatitis, and warts (Multimedia Appendix 3). In broad categories, 48% (333/704) were categorized as inflammatory diagnoses, 29% (203/704) infectious, 10% (69/704) neoplastic, 3% (21/704) diseases of vasculature, and 19% (73/704) other.

**Table 3:** Outcomes of consultations sent by nondermatologist providers (N=503).

Measures			Consultations, n (%)
**Time to** **initial** **response**			
	0-59 minutes	323 (64)	
	1-6 hours	120 (24)	
	6-12 hours	35 (7)	
	12-24 hours	8 (2)	
	24-48 hours	6 (1)	
	>48 hours	11 (2)	
**Time to final diagnosis or recommendation**			
	0-59 minutes	272 (54)	
	1-6 hours	144 (29)	
	6-12 hours	35 (7)	
	12-24 hours	13 (3)	
	24-48 hours	10 (2)	
	>48 hours	14 (3)	
	No final diagnosis or recommendation	15 (3)	
**Diagnosis provided^a^**			
	Single diagnosis	259 (52)	
	Multiple diagnoses	15 (3)	
No diagnosis made			229 (46)
Differential diagnosis provided^a^			159 (32)
**Dermatologist recommendations**			
	Local management	281 (56)	
	Dermatology referral	163 (32)	
	Referral to other specialist	15 (3)	
	Other	44 (9)	
Education provided			140 (28)

^a^Each patient could have single or multiple conditions presented by the consulting provider. Each of these conditions was considered separately by the evaluating dermatologist. The evaluating dermatologist could provide a single diagnosis, multiple diagnoses (at least 2), no diagnosis, and/or a differential diagnosis for any condition they determined was present.

### Satisfaction Survey of Providers Using WhatsApp for Teledermatology

**Figure 3:**
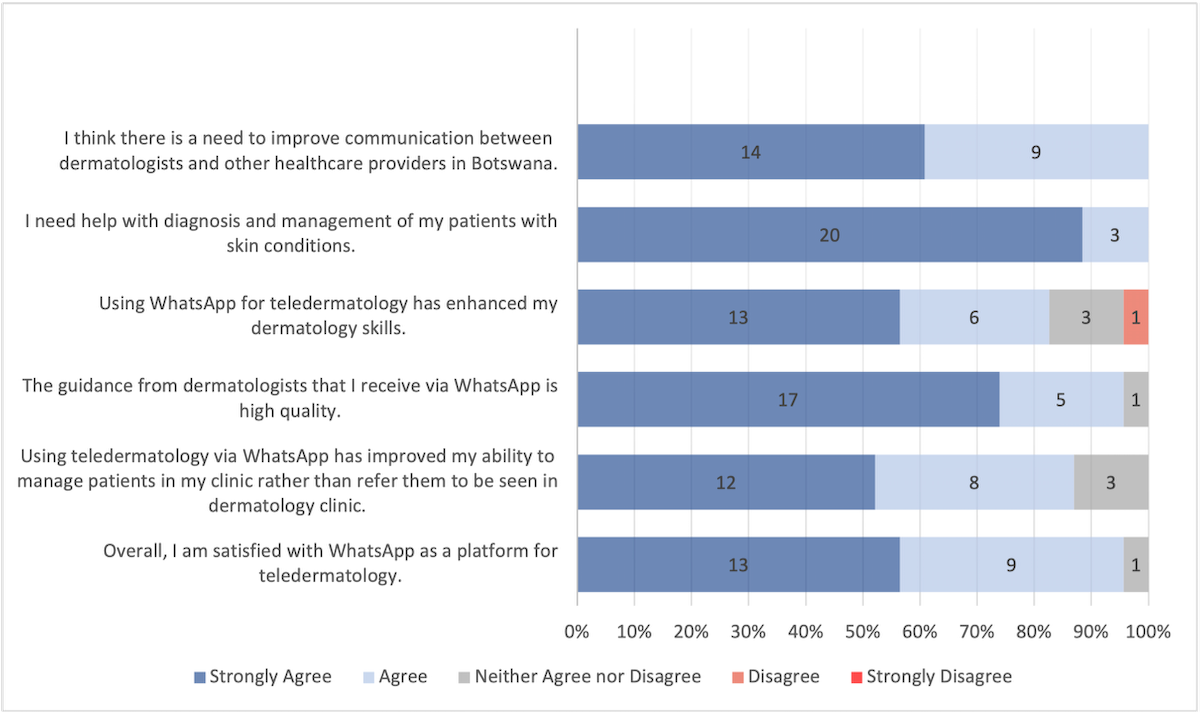
Responses to the Provider Satisfaction Survey questions assessing the overall utility of WhatsApp as a teledermatology platform.

A survey was sent out to approximately 150 health care providers, of which 15% (23/150) completed the survey (Multimedia Appendix 2). Demographics of survey respondents are shown in Multimedia Appendix 4. All respondents felt that there was a need for teledermatology, improved teledermatology education, and improved communication between dermatologists and other health care providers in Botswana (Figure 3). Most respondents (20/23, 87%) strongly agreed that they needed help with diagnosing and managing skin conditions, 83% (19/23) agreed that using WhatsApp for teledermatology enhanced their dermatology skills, and 87% (20/23) felt it improved their ability to manage patients in their own clinic to avoid referral. Most respondents (17/23, 74%) strongly felt that guidance received via WhatsApp was of high quality, and 96% (22/23) were satisfied with WhatsApp as a platform for teledermatology (Figure 3). The highest-rated features of using WhatsApp as a teledermatology platform included the ease of sending consults (21/23, 91%), having previous knowledge on how to use the application (20/23, 87%), and ease of asking follow-up questions (19/23, 83%) (Table 4).

In terms of privacy and security, only two-thirds (15/23, 65%) of respondents reported always obtaining consent from patients for photos to be sent via teledermatology. Of those who obtained consent, all obtained verbal instead of written consent. Nearly all respondents used a personal phone (21/23, 91%) or camera (1/23, 4%). A majority (14/23, 61%) kept these photos on a password protected device, but nearly one-third (9/23, 39%) did not or only occasionally did. Most respondents (19/23, 83%) were not concerned about privacy or security issues while using WhatsApp for teledermatology. Concerns reported included the possibility of hacking, forwarding photos, and inappropriate access by third parties (Table 4).

When asked about areas of improvement, respondents shared issues regarding the timing of responses, availability of consultants, and difficulty keeping case discussions organized when multiple separate patient consults were sent within 1 text thread. Another provider expressed concern about patients being able to obtain an in-person follow-up by a dermatologist when needed.

**Table 4:** Responses to the Provider Satisfaction Survey questions assessing specific features of WhatsApp as a teledermatology platform and patient data safety practices (N=23).

Questions		Responses, n (%)
**Which of the following features do you like about using WhatsApp for teledermatology? (Select all that apply)**		
	Easy to send consults	21 (91)
	I already have and know how to use the application	20 (87)
	Easy to ask follow-up questions	19 (83)
	Fast response times	14 (61)
	Doesn’t require a computer with Internet	10 (44)
	Easy to get patients urgently scheduled with dermatology clinic	11 (48)
**Do you obtain consent from patients for photos to be transmitted by teledermatology?**		
	Always	15 (65)
	Almost always	6 (26)
	Often	1 (4)
	Never	1 (4)
**How do you obtain consent from patients?**		
	Verbal	23 (100)
	Written	0 (0)
**What device do you use to send consults?**		
	Personal phone	21 (91)
	Work phone	3 (13)
	Personal camera	1 (4)
	Work camera	0 (0)
	Other	0 (0)
**Do you keep patient photos on a password-protected device?**		
	Yes	14 (61)
	No	7 (30)
	Sometimes	2 (9)
**Do you have concerns about the privacy and/or security of using WhatsApp for teledermatology?**		
	No	19 (83)
	Yes	4 (17)

## Discussion

### Principal Findings

This pilot study describes the use of WhatsApp, a popular text messaging app, as an informal teledermatology platform for consultation and education in Botswana and demonstrates that WhatsApp provides a rapid and well-received method of communication between dermatologists and other health care providers.

In our study, the most common use of WhatsApp by health care providers was to consult a dermatologist about a specific patient. Our results indicate that WhatsApp facilitates rapid discussion of dermatology cases, as dermatologists responded within 1 hour for the majority of consults. In addition, group messaging was utilized to provide a platform for simultaneous communication among a team of care providers to facilitate multidisciplinary care coordination, which has been a particular challenge in Botswana [6]. Overall, our study demonstrated that WhatsApp is being used as a direct line of communication between providers to promote care coordination, provide triage advice for life-threatening conditions, disseminate dermatology education, and allow for direct patient care when appropriate. This reinforces the previous conclusions of Littman-Quinn et al [5] that mHealth tools may offer a solution for improving access to specialty care in resource-limited settings by increasing access to specialty referrals, point-of-care information, and medical decision-making support for complex dermatologic cases.

The sustainability of teledermatology platforms has been a historical challenge in resource-limited countries [21]. Compared to previous formal telemedicine platforms that have not been successful in the long term in Botswana, WhatsApp has several attributes that increase its potential for sustainability: ease of use, free access on personal mobile devices, and no dedicated funding required to maintain it as a telemedicine platform [5,22]. To date, WhatsApp has been used for over 4 years as a teledermatology tool at Princess Marina Hospital in Botswana and has been increasing in popularity since its inception [5,22]. Around 90% of survey respondents indicated they valued the simplicity and familiarity of the application. In another low-resource areas in the Middle East, a survey illustrated a similarly high satisfaction rate with mHealth–based teledermatology, which was also attributed in part to feasibility [23].

One potential drawback of informal platforms such as WhatsApp is the lack of standardized consultation format, which allows for free-text submission of consults that may be incomplete or contain an inadequate amount of information. The ability to have real-time conversational exchanges can help overcome the lack of structured consults, though this can cause inefficiency. In this study, dermatologists asked clarifying questions in nearly 40% of the consultations. Regarding photo quality, only 20% of consults included photos that were considered low quality in our subjective assessment, primarily due to user errors such as poor lighting or blurriness rather than low resolution. Notably, the subjective rating of photographs did not always correlate with file size, suggesting that high quality photos could be obtained with low-tech mobile cameras. Future studies using validated methods to assess photo quality are needed to further explore this issue.

In this study, dermatologists were able to make a wide variety of skin diagnoses in over half of the consultations, indicating that the history and quality of photos in WhatsApp consultations could adequately support remote evaluation and diagnosis. Knowing whether telemedicine diagnoses are accurate is essential when considering the utility of providing or upscaling such services; however, we were unable to assess diagnostic accuracy in this small pilot study. Some studies have indicated that diagnoses made by teledermatology can be reliable and accurate [24], but data are lacking for teledermatology on mobile devices and in settings like Botswana [18]. In our opinion, common conditions like eczema, acne, and herpes simplex virus are often simple to diagnose via teledermatology and can be managed remotely by local providers. This can save time and costs for patients, providers, and the health care system. Moreover, using teledermatology for serious and life-threatening conditions, such as the 14 cases of Stevens Johnson Syndrome identified in this study, allows for same-day triaging to appropriate care that could save lives.

WhatsApp facilitated remote management in over half of the consultations in our study, reducing the need for an in-person consultation and potentially reducing the travel and cost burdens to patients and the health care system. Patients and providers were distributed widely across Botswana, and WhatsApp was able to successfully connect patients and providers across large distances, reaching urban and rural areas. Prior research has also shown that teledermatology can help decrease unnecessary health care spending and improve allocation of resources by reducing unnecessary referrals and outpatient visits [25]. Additionally, by reducing the number of patients that need to be seen in person, WhatsApp teledermatology consults have the potential to increase access to care for other patients with more severe skin conditions to be seen in dermatology clinics [26].

WhatsApp also has the potential to be used for provider education. In about one-third of consultations, the dermatologist provided education to complement management recommendations. Education is particularly valuable in resource-limited settings, where providers often lack access to clinical educational resources to assist in point-of-care decisions [27]. One-on-one, case-based education may help to empower providers to manage dermatologic conditions independently; however, WhatsApp has limitations when it comes to disseminating information broadly, which is important for education on a health systems level.

Our survey results showed that WhatsApp is a well-received and valuable resource for nondermatology providers. All but 1 respondent were satisfied with WhatsApp as a teledermatology platform, and many reported that it improved the quality of care they delivered. Respondents liked the familiarity of WhatsApp, which is consistent with WhatsApp being the predominant form of mobile communication in Botswana [20]. Other studies examining teledermatology and the use of mobile-health platforms in low-resource settings have shown similarly high levels of provider satisfaction [5,23,28,29].

When considering telemedicine, the privacy and security of shared patient information is extremely important. In teledermatology, many consultations include protected health information and photos of patients’ faces or sensitive body areas [5,6]. Most respondents in this study reported little to no concern about the security of images obtained and sent, and WhatsApp has multiple features to increase security to message transmission such as end-to-end encryption [30]. However, most providers took images on their personal phones. Nearly one-third stored images on devices that were not password protected, and almost one-third occasionally or rarely obtained patient consent to take photos to send to other providers. It has been reported that the sharing of medical photography between physicians on personal smartphones is generally accepted by patients, who may feel that the benefit of receiving timely, quality medical care outweighs the risks of data security from texting or emailing between physicians [31]. However, patient expectations may vary, and physicians should follow local laws and regulations regarding patient privacy. All telemedicine systems, and indeed all medical systems, carry some risk for patient privacy breaches, and some countries have additional guidelines to prevent accidental exposure of confidential information [22].

### Limitations

Our study has several limitations. Data collection was a manual process with only 1 author reading each conversation thread, increasing the risk for errors and subjectivity, particularly in terms of grading photos. Due to the retrospective nature of the study, the heterogeneity of information provided, and the nature of downloading WhatsApp messages, we were unable to accurately calculate the number of users and all patient demographics. In addition, the number of patients electronically visited was not able to be assessed given the lack of a medical record number or chart linked to each informal teledermatology consult. As previously discussed, this study did not measure accuracy of diagnoses made via WhatsApp, which would be required to measure the overall effectiveness of the platform for teledermatology. The survey was a subjective measurement of the perceived value of teledermatology, not based on a previously validated or reliable survey instrument. A limited number of questions were used to avoid participant burden and survey fatigue. Due to low response rate, survey results may not represent the opinions of all providers using WhatsApp for teledermatology. Reasons for the low response rate are unknown, but they may include the distribution of surveys by cellular messaging, the lack of incentive for participating, and that some providers messaged were no longer participating in WhatsApp teledermatology. Additionally, study findings may not be generalizable to other resource-limited settings due to various regional differences. Despite these limitations, this pilot study serves as an important baseline to inform future investigations of WhatsApp to include diagnostic accuracy, patient acceptability, health outcomes, and the development of standardized guidelines for provider exchange.

### Conclusions

Access to dermatology expertise remains a critically limited resource in Botswana. This study shows that there has been consistent and well-received use of WhatsApp for teledermatology in Botswana without dedicated funding, training, or equipment. The platform demonstrates a potential to support a variety of clinical purposes, such as patient consultations, triage and referral, multidisciplinary care coordination and point-of-care education. High satisfaction levels and an improvement in the ability to diagnose and manage a range of dermatologic conditions were evidenced by WhatsApp user feedback. Drawbacks identified include a lack of structured consultation format, potential security risks for patient information, and the inability to integrate consult information into a patient’s record. Despite these drawbacks, convenient, informal teledermatology platforms such as WhatsApp show promise in overcoming the logistical and sustainability challenges that have hampered teledermatology efforts in resource-limited settings. Further studies are needed to assess the effectiveness of WhatsApp and evaluate patient acceptability. The information gained from this study can serve as a baseline for future telemedicine studies and to inform the design and implementation of teledermatology in Botswana and other resource-limited countries.

## Appendix

Multimedia Appendix 1 Summary of data extraction and categorization methods used for analysis of WhatsApp communication threads.

Multimedia Appendix 2 Provider Satisfaction Survey.

Multimedia Appendix 3 Top ten most common conditions diagnosed by dermatologists via WhatsApp. Evaluating dermatologists made a total of 704 diagnoses and differential diagnoses which included 224 unique conditions. The numerical value and percentages in the table represent a portion of the 704 diagnoses and differential diagnoses.

Multimedia Appendix 4 Demographics of survey respondents.

## Abbreviations

BUP: Botswana-UPenn Partnership
HPI: history of present illness
IRB: institutional review board
mHealth: mobile health
PMH: Princess Marina Hospital
REDCap: research electronic data capture
