# Supplementary material for: Evaluation of WhatsApp as a Platform for Teledermatology in Botswana: Retrospective Review and Survey
Source: JMIR Dermatol. 2022 Jul 27;5(3):e35254. doi: 10.2196/35254 (PMC10334913; doi:10.2196/35254)
Supplement: Multimedia Appendix 1 [file derma_v5i3e35254_app1.docx]

Multimedia Appendix 1: Summary of data extraction and categorization methods used for analysis of WhatsApp communication threads.

| Category | Entry | Entry Options |
| --- | --- | --- |
| Communication categorization | Type of communication | Consultation from provider |
|  |  | Consultation from patient |
|  |  | Remote patient management |
|  |  | Patient follow up |
|  |  | Tele-triage |
|  |  | Multidisciplinary care coordination |
|  |  | Provider question |
|  |  | Incomplete communication |
| Sender information | Phone number | <free text> |
|  | Location | <free text> |
|  | Profession | <free text> |
| Patient demographics^a^ | Age | <free text> |
|  | Sex | Male |
|  |  | Female |
|  |  | Unknown |
|  | HIV status | Positive |
|  |  | Negative |
|  |  | Unknown |
| Patient history^a^ | History of present illness (one point for each)^b^ | Description of lesion |
|  |  | Location on body |
|  |  | Symptoms reported |
|  |  | Timing of onset |
|  |  | Change in appearance over time |
|  |  | Aggravating or alleviating factors |
|  |  | Prior treatments |
|  |  | Pertinent lab or imaging results |
| Photo quality parameters^a^ | File size | <50 kB |
|  |  | 50-100 kB |
|  |  | 100-150 kB |
|  |  | >150 kB |
|  | Image resolution | High (clear) [one point] |
|  |  | Low (blurry) [zero point] |
|  | Lighting | Adequate (achieves visualization of definition, texture, and detail) [one points] |
|  |  | Inadequate (does not achieve visualization of definition, texture, and detail) [zero points] |
|  | Relevant area | Captured (regional view that establishes location and provides general context of lesion described) [one point] |
|  |  | Not captured (image fails to establish location or provide context of lesion described) [zero points] |
|  | Overall subjective grade (based on cumulative points from image resolution, lighting, and relevant area categories) | Low (0-1 points) |
|  |  | Medium (2 points) |
|  |  | High (3 points) |
| Response time^a^ | Send date | MM/DD/YYYY |
|  | Time from initial message to initial response | 0-60 minutes |
|  |  | 1-6 hours |
|  |  | 6-12 hours |
|  |  | 12-24 hours |
|  |  | 24-48 hours |
|  |  | >48 hours |
|  | Time from initial message to final diagnosis or recommendation | 0-60 minutes |
|  |  | 1-6 hours |
|  |  | 6-12 hours |
|  |  | 12-24 hours |
|  |  | 24-48 hours |
|  |  | >48 hours |
|  |  | No final diagnosis or recommendation made |
| Dermatologist response and outcome^a^ | Was a diagnosis made or differential diagnosis provided? | No diagnosis made |
|  |  | Single diagnosis made |
|  |  | Multiple diagnoses made |
|  |  | Differential provided |
|  | Diagnosis(es) | <free text> |
|  | Differential diagnosis(es) | <free text> |
|  | Recommendation | Local management |
|  |  | Dermatology referral |
|  |  | Referral to other specialist |
|  |  | Other |
|  | Education provided^c^ | Yes |
|  |  | No |

^a^Data collected only from communications within the “consultations from provider” category.

^b^Historical information relevant to the patient's dermatologic complaint that was shared by the provider in their initial consult message. Information obtained after the dermatologist asked clarifying questions was not included.

^c^Educational information provided by the dermatologist in addition to diagnosis, differential diagnosis, and medical management.
