# Supplementary material for: Evaluation of WhatsApp as a Platform for Teledermatology in Botswana: Retrospective Review and Survey
Source: JMIR Dermatol. 2022 Jul 27;5(3):e35254. doi: 10.2196/35254 (PMC10334913; doi:10.2196/35254)
Supplement: Multimedia Appendix 2 [file derma_v5i3e35254_app2.docx]

Multimedia Appendix 2: Provider Satisfaction Survey

| Category | Question or Prompt | Entry Options |
| --- | --- | --- |
| Demographics | Where do you currently work? | <free text> |
|  | What is your current job position? | <free text> |
|  | What is your gender? | <free text> |
|  | Date of Birth | <free text> |
|  | Age | <free text> |
| WhatsApp Teledermatology Evaluation | I think that there is a need for teledermatology in Botswana. | *Select one of the following:*  Strongly Agree  Agree  Neither Agree nor Disagree  Strongly Disagree |
|  | I think there is a need for improved dermatology education in Botswana. | *Select one of the following:*  Strongly Agree  Agree  Neither Agree nor Disagree  Strongly Disagree |
|  | I think there is a need to improve communication between dermatologists and other healthcare providers in Botswana. | *Select one of the following:*  Strongly Agree  Agree  Neither Agree nor Disagree  Strongly Disagree |
|  | I need help with diagnosis and management of my patients with skin conditions | *Select one of the following:*  Strongly Agree  Agree  Neither Agree nor Disagree  Strongly Disagree |
|  | Using WhatsApp for teledermatology has enhanced my dermatology skills. | *Select one of the following:*  Strongly Agree  Agree  Neither Agree nor Disagree  Strongly Disagree |
|  | The guidance from dermatologists that I receive via WhatsApp is high quality. | *Select one of the following:*  Strongly Agree  Agree  Neither Agree nor Disagree  Strongly Disagree |
|  | Using teledermatology via WhatsApp has improved my ability to manage patients in my clinic rather than refer them to be seen in dermatology clinic. | *Select one of the following:*  Strongly Agree  Agree  Neither Agree nor Disagree  Strongly Disagree |
|  | Overall, I am satisfied with WhatsApp as a platform for teledermatology. | *Select one of the following:*  Strongly Agree  Agree  Neither Agree nor Disagree  Strongly Disagree |
|  | Which of the following features do you like about using WhatsApp for teledermatology? | *Select all that apply:*  I already have and know how to use the application  Easy to send consults  Fast response times  Doesn’t require a computer with internet  Easy to ask follow up questions  Easy to get patients urgently scheduled with dermatology clinic |
|  | Is there anything you don’t like or would change about using WhatsApp for teledermatology? | <free text> |
|  | Do you obtain consent from patients for photos to be transmitted by teledermatology? | *Select one of the following:*  Always  Almost always  Often  Never |
|  | How do you obtain consent from patients? | *Select one of the following:*  Written  Verbal  Other |
|  | If other, please specify | <free text> |
|  | What device do you use to send consults? | *Select all that apply:*  Personal phone  Work phone  Personal camera  Work camera  Other |
|  | If other method of consent, please specify | <free text> |
|  | Do you keep patient photos on a password protected device? | *Select one of the following:*  Yes  No  Sometimes |
|  | Do you have concerns about the privacy and/or security of using WhatsApp for teledermatology? | *Select one of the following:*  Yes  No |
|  | If yes, please specify the concern | <free text> |
