# Supplementary material for: Evaluation of WhatsApp as a Platform for Teledermatology in Botswana: Retrospective Review and Survey
Source: JMIR Dermatol. 2022 Jul 27;5(3):e35254. doi: 10.2196/35254 (PMC10334913; doi:10.2196/35254)
Supplement: Multimedia Appendix 3 [file derma_v5i3e35254_app3.docx]

Multimedia Appendix 3: Top ten most common conditions diagnosed by dermatologists via WhatsApp. Evaluating dermatologists made a total of 704 diagnoses and differential diagnoses which included 224 unique conditions. The numerical value and percentages in the table represent a portion of the 704 diagnoses and differential diagnoses.
