# Supplementary material for: Evaluation of WhatsApp as a Platform for Teledermatology in Botswana: Retrospective Review and Survey
Source: JMIR Dermatol. 2022 Jul 27;5(3):e35254. doi: 10.2196/35254 (PMC10334913; doi:10.2196/35254)
Supplement: Multimedia Appendix 4 [file derma_v5i3e35254_app4.docx]

Multimedia Appendix 4: Demographics of survey respondents.

| Characteristic | Respondents (N=23) |
| --- | --- |
|  | n (%) |
| Gender |  |
| Female | 13 (58%) |
| Male | 10 (42%) |
| Current job position |  |
| Medical officer | 14 (61%) |
| Physician | 8 (35%) |
| Resident | 1(4%) |
| Location |  |
| Urban city | 13 (57%) |
| Rural village | 8 (35%) |
| Unknown | 2 (9%) |
